# Engineering of Thermoelectric Composites Based on Silver Selenide in Aqueous Solution and Ambient Temperature

**DOI:** 10.1021/acsaelm.3c00055

**Published:** 2023-05-05

**Authors:** Bingfei Nan, Mengyao Li, Yu Zhang, Ke Xiao, Khak Ho Lim, Cheng Chang, Xu Han, Yong Zuo, Junshan Li, Jordi Arbiol, Jordi Llorca, Maria Ibáñez, Andreu Cabot

**Affiliations:** † Catalonia Institute for Energy Research−IREC, Sant Adrià del Besòs, Barcelona 08930, Spain; ‡ Departament d′Enginyeria Electrònica i Biomèdica, Universitat de Barcelona, Barcelona 08028, Catalonia, Spain; § School of Physics and Microelectronics, Zhengzhou University, Zhengzhou 450052, China; ∥ Department of Materials Science and Engineering, Pennsylvania State University, State College, Pennsylvania 16802, United Sates; ⊥ Institute of Zhejiang University−Quzhou, 99 Zheda Road, Quzhou 324000, Zhejiang, P.R. China; # College of Chemical and Biological Engineering, Zhejiang University, 38 Zheda Road, Hangzhou 310007, Zhejiang, P.R. China; □ Institute of Science and Technology Austria (ISTA), Am Campus 1, Klosterneuburg 3400, Austria; ○ School of Materials Science and Engineering, Beihang University, Beijing 100191, China; △ Catalan Institute of Nanoscience and Nanotechnology (ICN2), Campus UAB, Bellaterra, Barcelona 08193, Catalonia, Spain; ■ Istituto Italiano di Tecnologia, Via Morego 30, Genova 16163, Italy; ● Institute for Advanced Study, Chengdu University, Chengdu 610106, China; ▲ ICREA, Pg. Lluís Companys 23, Barcelona 08010, Catalonia, Spain; ▽ Institute of Energy Technologies, Department of Chemical Engineering and Barcelona Research Center in Multiscale Science and Engineering, Barcelona East School of Engineering, Universitat Politècnica de Catalunya, Barcelona 08019, Catalonia, Spain

**Keywords:** thermoelectricity, silver selenide, aqueous synthesis, bismuth sulfide, composite

## Abstract

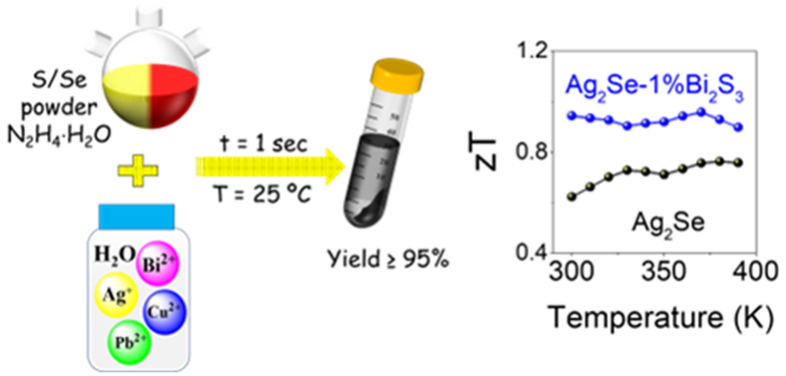

The direct, solid state, and reversible conversion between heat and electricity using thermoelectric devices finds numerous potential uses, especially around room temperature. However, the relatively high material processing cost limits their real applications. Silver selenide (Ag_2_Se) is one of the very few n-type thermoelectric (TE) materials for room-temperature applications. Herein, we report a room temperature, fast, and aqueous-phase synthesis approach to produce Ag_2_Se, which can be extended to other metal chalcogenides. These materials reach TE figures of merit (*zT*) of up to 0.76 at 380 K. To improve these values, bismuth sulfide (Bi_2_S_3_) particles also prepared in an aqueous solution are incorporated into the Ag_2_Se matrix. In this way, a series of Ag_2_Se/Bi_2_S_3_ composites with Bi_2_S_3_ wt % of 0.5, 1.0, and 1.5 are prepared by solution blending and hot-press sintering. The presence of Bi_2_S_3_ significantly improves the Seebeck coefficient and power factor while at the same time decreasing the thermal conductivity with no apparent drop in electrical conductivity. Thus, a maximum *zT* value of 0.96 is achieved in the composites with 1.0 wt % Bi_2_S_3_ at 370 K. Furthermore, a high average *zT* value (*zT*_ave_) of 0.93 in the 300–390 K range is demonstrated.

## Introduction

Thermoelectric (TE) devices allow for direct, solid state, and reversible conversion between heat and electricity.^1−4^ TE devices can harvest heat from the ambient environment, potentially increasing the efficiency of a plethora of processes. They also allow precise control of the temperature and effective cooling of hot spots. However, their real-world applications are limited to several niche markets due to their relatively low cost-effectiveness. The high cost of TE devices is related to the use of scarce elements such as Te, the need for high-temperature or vacuum-based processes for the synthesis of TE materials, and the quasimanual manufacturing of TE modules. Alternative printing technologies are being developed worldwide, but the use of organic solvents for the synthesis of the materials and/or the ink formulation is still a major drawback to the environmentally friendly and low-cost processing of TE devices. On top of the high cost, the energy conversion efficiency of TE devices is relatively low. The energy conversion efficiency of a TE material is determined by a dimensionless figure of merit
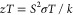
1where *S*, σ, κ, and *T* are the Seebeck coefficient (μV K^–1^), electrical conductivity (S m^–1^), thermal conductivity (W m^–1^ K^–1^), and absolute temperature (K), respectively. κ includes the electronic thermal conductivity (*κ*_e_) and lattice thermal conductivity (*κ*_L_):
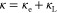
2Besides, S^2^σ is defined as the power factor (PF). A good TE material is thus characterized by high S and σ, and low κ values.

Silver selenide (Ag_2_Se) is one of the very few TE materials suitable for use at ambient temperature, where it is characterized by relatively low thermal conductivity and high electrical conductivity.^5^ Ag_2_Se is an n-type semiconductor with a narrow band gap (*E*_g_ = 0.07 eV at 0 K). It exists in two stable phases, the low-temperature orthorhombic β-phase and the high-temperature cubic α-phase, with a transition temperature of around 407 K.^6−8^ Numerous approaches exist for the synthesis of Ag_2_Se. Among them, the commonly reported solid-state preparation strategy is based on reacting the two elements, Ag and Se, at high temperatures^6,9−15^ or using high-energy ball milling.^8,16^ As an example, Chen et al. developed a porous Ag_2_Se with hierarchical structures via a wet mechanical alloying process. Using this approach, a low lattice thermal conductivity of ∼0.35 W m^–1^ K^–1^ and a zT of ∼0.7 were obtained at 300 K.^8^ Besides, Ag_2_Se is also produced by vacuum-based technologies such as magnetron sputtering.^17^ Various chemical synthetic methods have also been reported for the production of silver chalcogenides and particularly Ag_2_Se particles, including colloidal,^18−21^ hydrothermal,^22,23^ and microwave-assisted^24^ methods. In some cases, aqueous solutions have been used.^5,25,26^ As an example, Xiao et al. synthesized Ag_2_Se nanocrystals via a colloidal method, reaching a maximum *zT* value of 0.23 at the phase transition temperature of around 408 K.^19^ Wang et al. reported a general aqueous synthesis of nano/microscale binary silver chalcogenides (Ag_2_X, X = S, Se, Te) based on the reaction of Na_2_S/NaHSe/NaHTe and AgNO_3_ aqueous solution at the water boiling temperature. The molar ratios of Ag^+^/X^2–^ were adjusted from 2:1 to 2:1.1 and the maximum *zT* value of the resulting Ag_2_Se pellet was 0.84 at 380 K.^25^

The TE properties of pristine Ag_2_Se can be improved through extrinsic and intrinsic doping. Li et al. reported a hydrothermal solution route using ethylene glycol and glycerol as solvents to prepare Ag_2_Se at 180 °C, reaching a maximum *zT* of 0.7 at 317 K for Ag_2_Se, and up to 0.9 at 300 K when adding 0.1 wt % Sn doping at Ag sites.^27^ Variations in the stoichiometric ratio of silver to selenium were also investigated to control the concentration of free carriers, showing an obvious effect on the TE performance.^11,16,17^ In this direction, Jood et al. introduced an anion excess (≤1% of Se or S) into Ag_2_Se obtaining a notable improvement in carrier mobility and zT values up to ∼1.0 in the temperature range of 300–375 K.^13,14^ Another important approach to improving Ag_2_Se performance is to combine it with small amounts of other materials into nanocomposites.^28−31^ As an example, Ballikaya et al. added Cu_2_Se nanoinclusions in Ag_2_Se to improve the TE performance and thermal stability.^32^ Lim et al. obtained a high *zT* of 0.89 at 343 K through the simple blending of Ag_2_Se with Te nanorods.^18^ Besides, carbon nanotubes were also used as an effective nanofiller for enhancing the TE performance of Ag_2_Se.^5,23^

Bismuth sulfide (Bi_2_S_3_) is an n-type semiconductor composed of relatively abundant, nontoxic, and low-cost elements.^33^ Bi_2_S_3_ has poor zT values at ambient temperature, because of moderate electrical conductivity. However, it is characterized by high Seebeck coefficients (ca. −400 μV/K) and low thermal conductivities. Bi_2_S_3_ has been used as a doping phase to promote the TE properties of some TE materials, including Cu_1.8_S^34^ and Bi_2_Te_2.7_Se_0.3._^35^

Herein, we detail a facile, rapid, room temperature, and aqueous-based general approach to producing highly crystalline Ag_2_Se. Besides, a series of Ag_2_Se-x wt % Bi_2_S_3_ (x = 0, 0.5, 1.0 and 1.5) nanocomposites is produced by solution-blending and hot pressing. Interestingly, the incorporation of Bi_2_S_3_ results in a significant increase in the Seebeck coefficient. Furthermore, the optimized composition shows low thermal conductivity and a record-high *zT* of 0.96 at 370 K.

## Experimental Section

### Materials

Silver(I) nitrate (AgNO_3_, 99.9+%), copper(II) nitrate trihydrate (Cu(NO_3_)_2_·3H_2_O, 99%), lead(II) nitrate (Pb(NO_3_)_2_, 99+%), and hydrazine hydrate (N_2_H_4_·H_2_O, 64%) were supplied by Fisher Scientific. Selenium powder (Se, 200 mesh, ≥ 99.5% trace metals basis), bismuth nitrate pentahydrate (Bi(NO_3_)_3_·5H_2_O, ≥ 99.99%), thioacetamide (TAA, ≥ 99.0%), and nitric acid (HNO_3_, 68%) were purchased from Sigma-Aldrich. All chemicals were used without further purification using standard solution synthesis procedures.^36,37^

### Synthesis of Silver Selenide

In a typical synthetic method, 0.8494 g of AgNO_3_ was dissolved into 10 mL of deionized water (DIW). In parallel, a Se precursor solution was prepared by adding 0.2078 g of Se powder to 5 mL of N_2_H_4_·H_2_O. The AgNO_3_ aqueous solution was then injected at ambient temperature into the Se solution, where a black precipitate was immediately formed. The product was collected by centrifugation and washed using DIW and ethanol three times. The final product was dried and stored in an Ar-filled glovebox.

### Synthesis of Metal Chalcogenide (MX)

For the production of other binary metal chalcogenides (MX, M = Cu, Pb, Bi; X = S, Se) a similar synthesis strategy was adopted. The detailed parameters are shown in Table S1. It is worth mentioning that SnS and SnSe can also be prepared by the same approach in an alkaline water environment (e.g., sodium hydroxide aqueous solution), but they require a longer reaction time (ca. 1 h) at ambient temperature.

### Synthesis of Bismuth Sulfide

Bi_2_S_3_ was synthesized in an aqueous solution following an alternative procedure inspired by previous publications.^38,39^ Briefly, TAA (0.510 g) was dissolved in 160 mL of DIW with rapid stirring. At the same time, Bi(NO_3_)_3_·5H_2_O (1.584 g) was added to 20 mL of 0.4 M HNO_3_ aqueous solution, and then it was added drop by drop to the TAA solution. The mixture was reacted for 15 h at room temperature with continuous strong stirring. The coarse product solution was washed with DIW and ethanol five times. Finally, it was dried and stored in an Ar-filled glovebox.

### Nanopowder Blend and Consolidation

Ag_2_Se/Bi_2_S_3_ composite powders were produced by blending the proper ratio of particles of the two materials in solution under ultrasonication for 1 h. Next, the dried blended powders were placed in a furnace and annealed at 250 °C for 1 h in an Ar/H_2_ flow. The annealed powders were loaded into a graphite die (Ø 10 mm × 10 mm cylinders) and hot-pressed for 5 min at 50 MPa and 250 °C inside an argon-filled glovebox. The hot-pressed pellets were then polished and used for TE characterization.

## Results and Discussion

**Figure 1:**
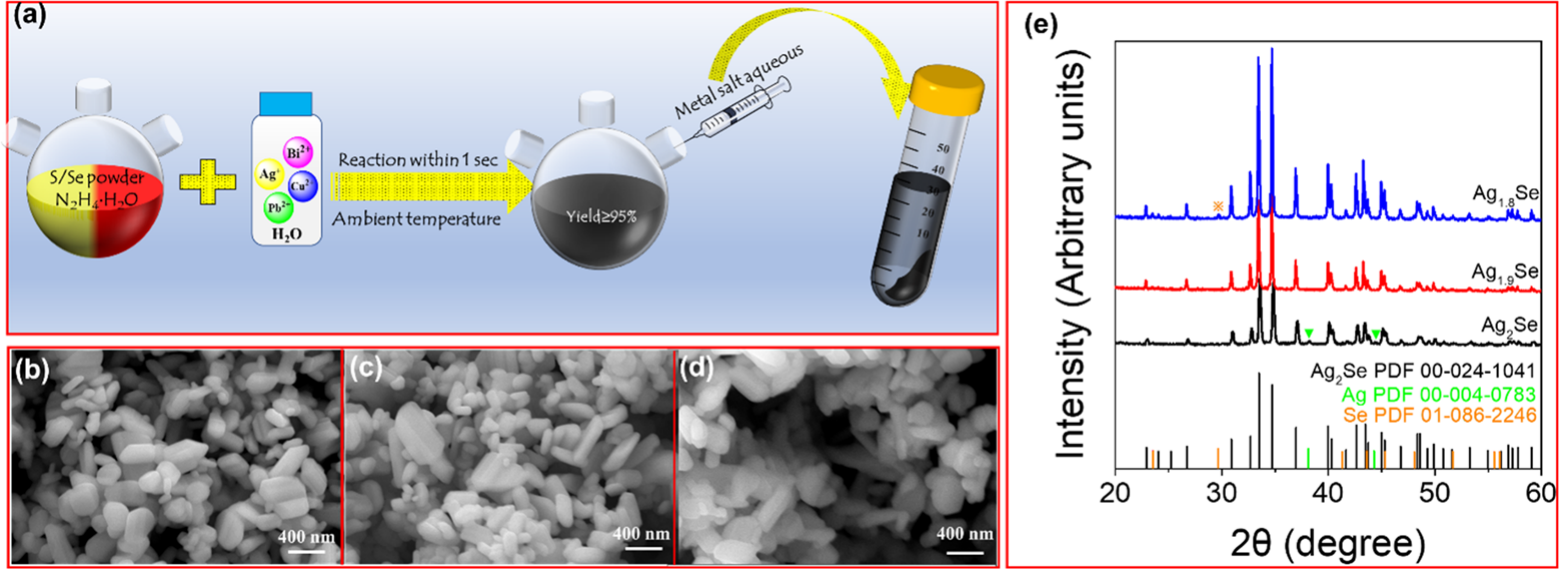
(a) Schematic illustration of the synthesis of binary metal chalcogenides (MX; M = Ag, Cu, Pb, Bi; X = S, Se). (b–d) SEM images of Ag_2_Se produced from AgNO_3_:Se molar ratios of (b) 2:1, (c) 1.9:1, and (d) 1.8:1. (e) XRD patterns of Ag_2_Se.

Figure 1a shows a schematic illustration of the aqueous and ambient temperature synthesis process used to produce binary metal chalcogenides (MX; M = Ag, Cu, Pb, Bi; X = S, Se). The MX chalcogenide is produced by the reaction of the zerovalent chalcogen (X^0^) powder with N_2_H_4_·H_2_O to form X^2–^ and the immediate reaction of such anions with the metal cations in the metal salt solution. In this way, Ag_2_S, Ag_2_Se, CuS, Cu_2_S, Cu_2_Se, Bi_2_S_3_, Bi_2_Se_3_, PbS, and PbSe particles, which through proper processing can be used in a plethora of different applications,^40−48^ were easily and rapidly obtained. Figure 1b–d displays scanning electron microscopy (SEM) images of Ag_2_Se produced from different AgNO_3_:Se molar ratios; 2:1, 1.9:1, and 1.8:1. Ag_2_Se particles are characterized by elongated shapes, an average size of a few hundred nanometers, and high crystallinity, as observed by X-ray diffraction (XRD, Figure 1e). XRD patterns show the obtained Ag_2_Se to have an orthorhombic crystallographic phase (PDF 00–024–1041) with lattice parameters *a* = 4.333 Å, *b* = 7.062 Å, and *c* = 7.764 Å. At an AgNO_3_:Se molar ratio of 2:1, a few impurity peaks at 38.2° and 44.3° can be indexed with the cubic Ag phase (PDF 00–004–0783). At a AgNO_3_/Se molar ratio of 1.8:1, a new peak at 29.6° is ascribed to the hexagonal Se phase (PDF 01–086–2246), indicating that the excess Se was not fully incorporated into the Ag_2_Se lattice. At a AgNO_3_:Se molar ratio of 1.9:1, XRD patterns show pure-phase Ag_2_Se, with no crystalline impurities. Thus, we chose this precursor molar ratio to prepare the material to be further characterized and used to produce Ag_2_Se/Bi_2_S_3_ composites. At this AgNO_3_:Se molar ratio of 1.9:1, energy-dispersive X-ray spectroscopy (EDX) analysis shows the Ag:Se atomic ratio in the final Ag_2_Se particles to be 2.2 (Figure S1). However, for the sake of convenience, we denote the silver selenide as Ag_2_Se. The SEM images, To demonstrate the versatility of the synthesis approach here reported, EDX data and XRD patterns of other MX (M = Ag, Cu, Pb, and Bi, X = S and Se) are displayed in Figure S2, Table S2, and Figure S3, respectively.

**Figure 2:**
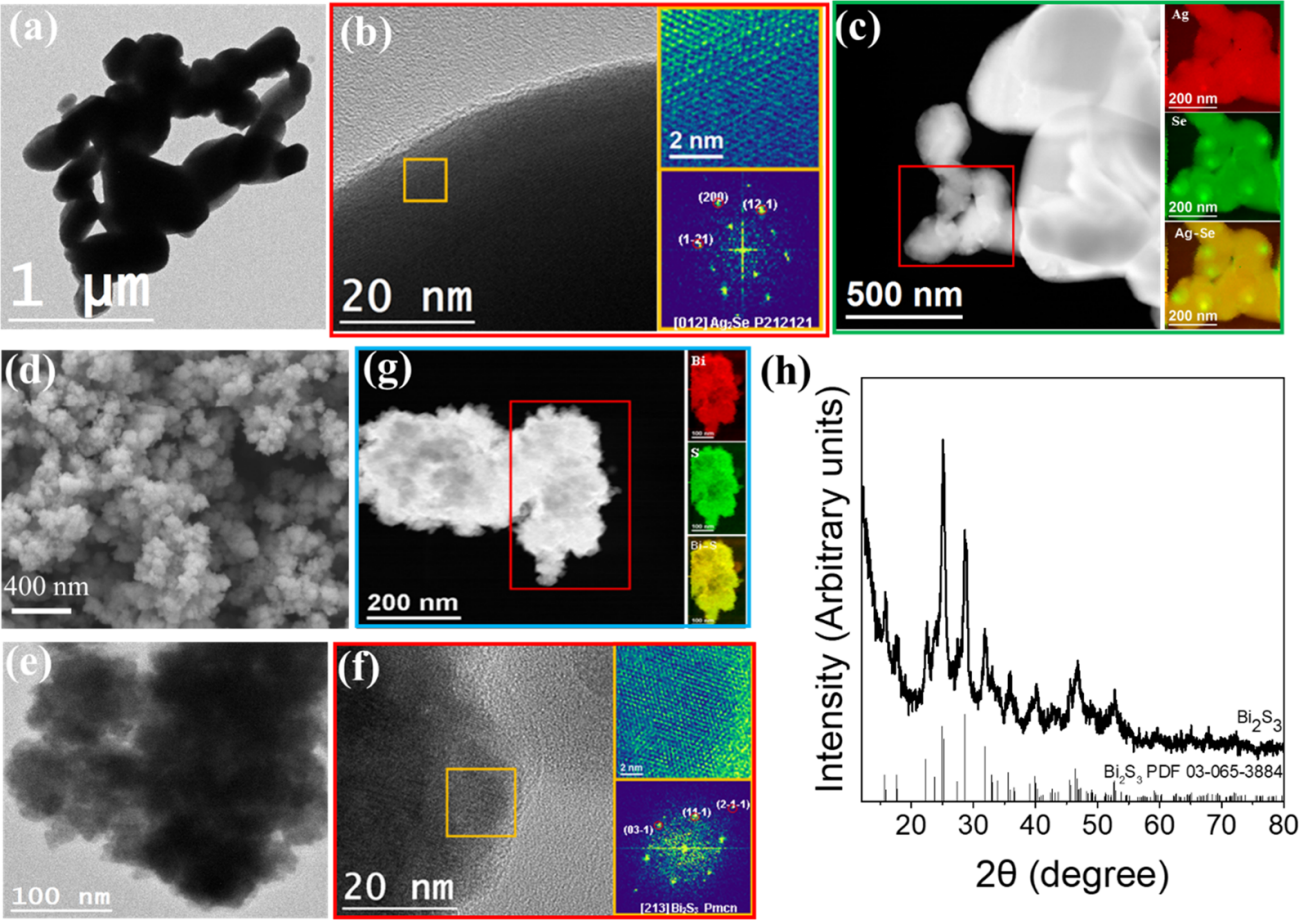
Structural and Chemical Characterization of Ag_2_Se and Bi_2_S_3_. (a) TEM micrograph of Ag_2_Se. (b) HRTEM micrograph of Ag_2_Se, detail of the orange squared region, and its corresponding power spectrum. (c) EELS chemical composition maps from the red square area of the STEM micrograph of Ag_2_Se. Individual Ag M4,5-edges at 367 eV (red), Se M1-edges at 232 eV (green), and composites of Ag–Se. (d) SEM image of Bi_2_S_3_. (e) TEM and (f) HRTEM micrograph of Bi_2_S_3_, detail of the orange square region, and its corresponding power spectrum. From the crystalline domain, the Bi_2_S_3_ lattice fringe distances were measured to be 0.351 0.354, and 0.193 nm, at 66.70° and 96.72°, which could be interpreted as the orthorhombic Bi_2_S_3_ phase visualized along its [213] zone axis. (g) EELS chemical composition maps from the red square area of the STEM micrograph of Bi_2_S_3_. Individual Bi N4,5-edges at 440 eV (red), S L2,3-edges at 165 eV (green), and composites of Bi–S. (h) XRD pattern of Bi_2_S_3_.

Figure 2a shows a general view bright field transmission electron microscopy (TEM) image of produced Ag_2_Se. Figure 2b shows a high-resolution HRTEM micrograph from an Ag_2_Se particle and its corresponding power spectrum revealing an orthorhombic crystal phase (space group *P*2_1_2_1_2_1_) with *a* = 4.334 Å, *b* = 7.070 Å, *c* = 7.774 Å. The high-angle annular dark field (HAADF) scanning TEM (STEM) micrographs and electron energy loss spectroscopy (EELS) composition maps of Ag_2_Se particles show a homogeneous distribution of both elements (Figure 2c).

Bi_2_S_3_ particles were also synthesized in an aqueous solution at ambient temperature. Figure 2d,e shows representative SEM and TEM images of the obtained product. Bi_2_S_3_ particles were highly polycrystalline and presented a flowerlike morphology. Crystallites had an average size of ca. 10 nm. EDX analysis showed the atomic ratio of Bi to S to be consistent with stoichiometric Bi_2_S_3_. HRTEM analysis showed the particle crystal structure to agree with the Bi_2_S_3_ orthorhombic phase (space group = *Pmcn*) with *a* = 3.9810 Å, *b* = 11.1470 Å, and *c* = 11.3050 Å (Figure 2f). EELS compositional maps showed a homogeneous distribution of Bi and S (Figure 2g). Besides, XRD data confirmed the orthorhombic phase (PDF 03–065–3884) of the Bi_2_S_3_ particles (Figure 2h).

**Figure 3:**
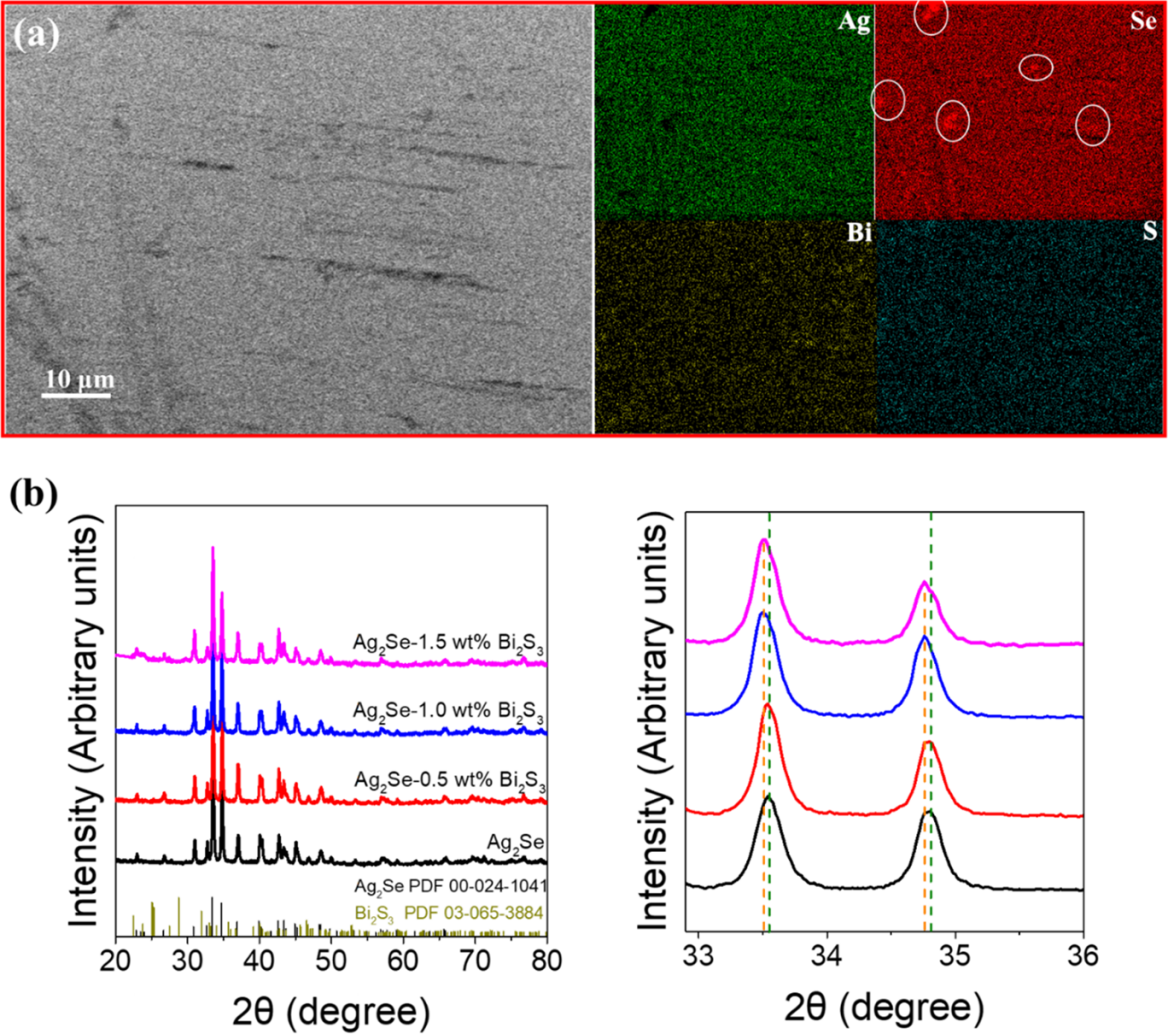
(a) SEM image of the polished Ag_2_Se-1.0 wt % Bi_2_S_3_ pellet and corresponding EDX compositional maps of Ag, Se, Bi, and S (Se-rich regions marked with white circles). (b) XRD patterns of consolidated Ag_2_Se-*x* wt % Bi_2_S_3_ pellets.

Ag_2_Se/Bi_2_S_3_ composites were produced by blending the proper ratio of particles in solution and hot pressing the resulting dried powder at 50 MPa and 250 °C inside an argon-filled glovebox (see the Experimental section for details). An SEM image of the polished surface of the Ag_2_Se-1.0 wt % Bi_2_S_3_ composite and its corresponding EDX elemental maps are shown in Figure 3a. Besides, the morphology of a fractured Ag_2_Se-1.0 wt % Bi_2_S_3_ sample and its compositional map and EDX compositions of all fractured pellets are shown in Figure S4 and Table S3. An overall homogeneous distribution of the constituent elements, including S and Bi, is observed, denoting atomic doping of the Ag_2_Se with these two elements. Only some small Se-rich inhomogeneities can be found, as marked with white circles. Figure 3b shows the XRD patterns of the hot-pressed Ag_2_Se-*x* wt % Bi_2_S_3_ composites, which can be indexed with the orthorhombic Ag_2_Se phase (PDF 00–024–1041). As the Bi_2_S_3_ content increases, the XRD peaks shift slightly to lower angles, indicating an expansion of the lattice associated with the partial substitution of Ag^+^ ions (0.67 Å) by Bi^3+^ ions (1.03 Å) with a larger ionic radius.^49^ However, when the Bi_2_S_3_ content exceeds 1.0 wt %, the XRD peaks no longer shift due to the limited solubility of Bi^3+^ in the matrix. No impurity XRD peaks and particularly Bi_2_S_3_ peaks were detected, indicating notable alloying of Bi_2_S_3_ with Ag_2_Se.^34^ The density of the composite slightly decreases with the increase of the Bi_2_S_3_ fraction due to the lower density of Bi_2_S_3_ (∼6.78 g/cm^3^) compared with Ag_2_Se^35^ but all samples reach relative densities above 90% (Table S4).

**Figure 4:**
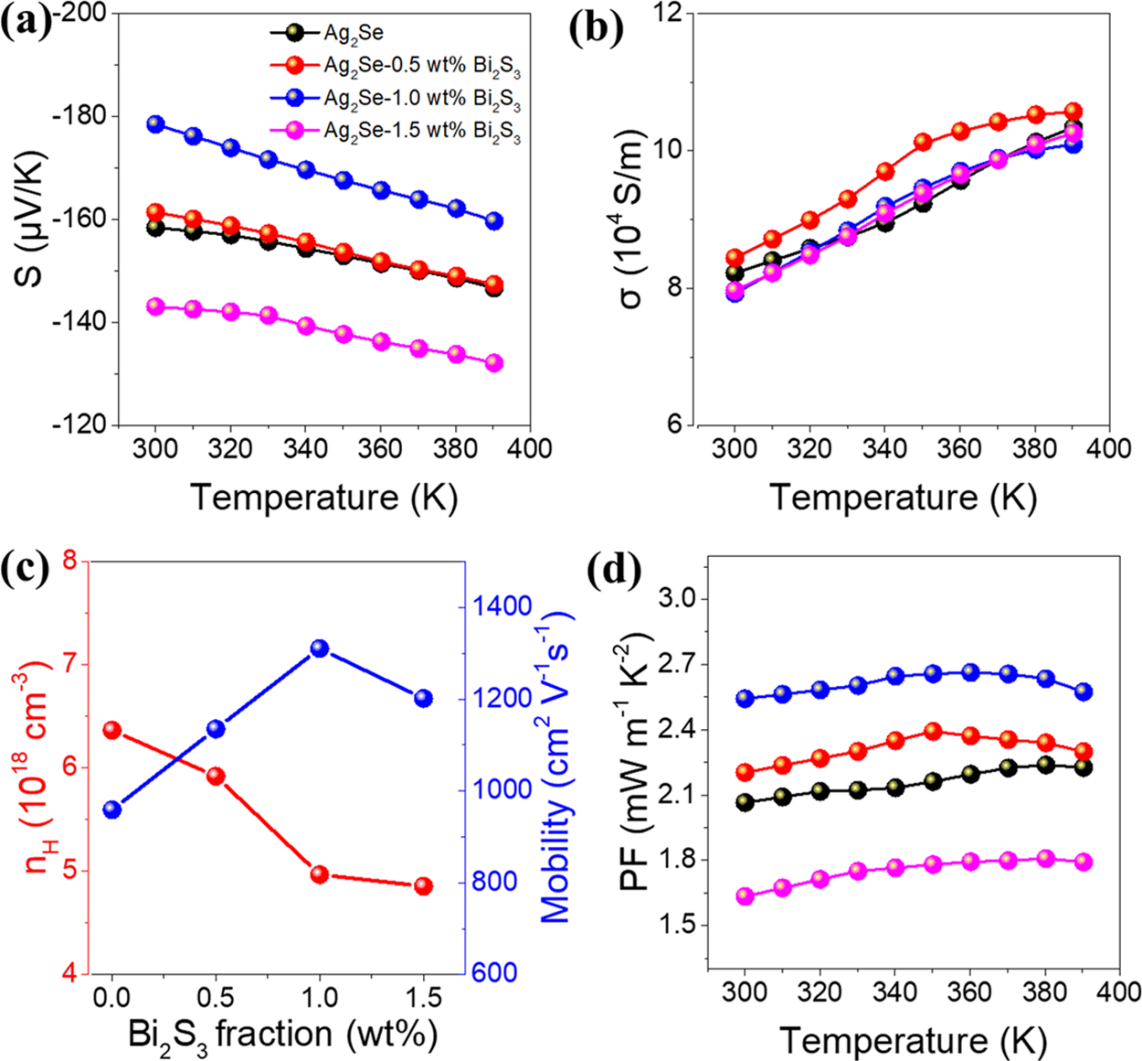
Temperature dependence of (a) Seebeck coefficient and *S*. (b) electrical conductivity, σ. (c) Hall carrier concentration (*n*_H_) and mobility (*μ*_H_) at room temperature. (d) Power factor PF of Ag_2_Se-*x* wt % Bi_2_S_3_.

The temperature dependence of the Seebeck coefficient (*S*) of the different composites is shown in Figure 4a. All samples show n-type semiconducting behavior with negative S values. The absolute values of S monotonously decrease with temperature over the entire measured range. The *S* of the pure Ag_2_Se sample reaches up to −158.4 μV/K at 300 K and decreases to −146.7 μV/K at 390 K. With the introduction of Bi_2_S_3_, the absolute *S* values increased significantly reaching up to −178.5 μV/K at 300 K for the Ag_2_Se-1.0 wt % Bi_2_S_3_ pellet.

As displayed in Figure 4b, the temperature dependence of the electrical conductivity (σ) of the different composites shows a typical nondegenerate semiconductor characteristic with σ monotonously increasing with temperature. Relatively similar σ values were obtained for the different doping composites.

The charge carrier concentration (*n*_H_) and mobility (*μ*_H_) as a function of the Bi_2_S_3_ amount were measured by Hall and are displayed in Figure 4c. As the concentration of Bi_2_S_3_ increases, the *n*_*H*_ for Ag_2_Se-*x* wt % Bi_2_S_3_ exhibits a moderate decrease (6.4 × 10^18^ cm^–3^ for pure Ag_2_Se and 4.9 × 10^18^ cm^–3^ for Ag_2_Se-1.5 wt % Bi_2_S_3_). In contrast, the *μ*_H_ for Ag_2_Se-*x* wt % Bi_2_S_3_ samples first rises and then decreases gradually with rising Bi_2_S_3_ concentration. In detail, the *μ*_H_ is 958.9 cm^2^ V^–1^ s^–1^ for the pure Ag_2_Se sample, and the largest value of 1310.4 cm^2^ V^–1^ s^–1^ is found as Bi_2_S_3_ amount increases to 1.0 wt %. A further increase of Bi_2_S_3_ doping concentration to 1.5 wt % reduces the *μ*_H_ of Ag_2_Se-*x* wt % Bi_2_S_3_ samples.

The increase in the absolute value of the Seebeck coefficient with the introduction of Bi_2_S_3_ is in part associated with the decrease in the charge carrier concentration. Previous studies also demonstrate an increase in the absolute value of the Seebeck coefficient and also the charge carrier mobility with the partial replacement of Se with S.^13,50^ Besides, the Bi doping within the Ag_2_Se lattice expands the lattice, as observed by XRD, which according to previous publications could increase the density of states near the Fermi level,^51−53^ thereby further enhancing the Seebeck coefficient of the Ag_2_Se-based materials. At too high, Bi_2_S_3_ precipitates are found as a secondary phase inside the Ag_2_Se matrix, which reduces the charge carrier mobility and the absolute value of the Seebeck coefficient. This reduction may be related to a higher bipolar contribution associated with the preferential scattering of electrons over holes at the Ag_2_Se/Bi_2_S_3_ interphase owing to the upward band bending generated at the Ag_2_Se side. It is therefore crucial to maintain the Bi_2_S_3_ content below 1% to achieve an optimal thermoelectric performance in Ag_2_Se–Bi_2_S_3_ composites. Notice that a similar evolution of the Seebeck coefficient with dopant concentration, first increasing and later decreasing at higher dopant concentrations, has been reported in other systems, and diverse mechanisms have been reported.^54−57^ Besides, previous studies have also shown increased Seebeck coefficients without affecting the electrical conductivity.^58−60^ The preserved electrical conductivity of Ag_2_Se after being mixed with Bi_2_S_3_ is attributed to the notable increase in charge carrier mobility, which compensates for the moderate decrease in charge carrier concentration.

Figure 4d shows the power factor (PF, S^2^σ) of Ag_2_Se-*x* wt % Bi_2_S_3_ samples as a function of temperature. For the pure Ag_2_Se sample, the PF slightly increases, from 2.06 to 2.24 mW m^–1^ K^–2^ over 300–390 K. The PF of the Ag_2_Se −1.0 wt % Bi_2_S_3_ composite is significantly larger, reaching up to 2.66 mW m^–1^ K^–2^ at 360 K. Notice also that the Ag_2_Se-1.0 wt % Bi_2_S_3_ pellet exhibits good stability even after multiple tests (Figure S5).

**Figure 5:**
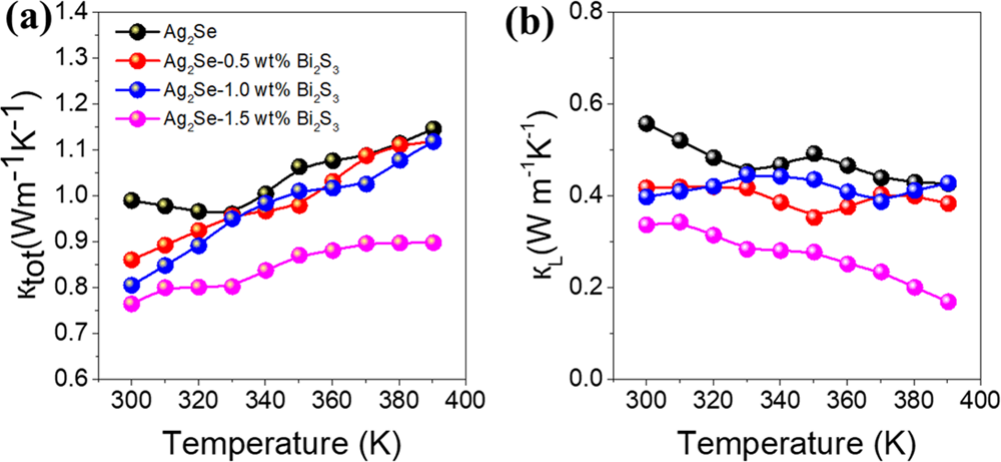
Thermal conductivity of (a) total thermal conductivity, κ_tot_. (b) Lattice thermal conductivity κ_L_.

The experimental thermal diffusivities, α, are presented in Table S4. The measured heat capacities, *C*_p_, and the limit *C*_p_ calculated by the Dulong–Petit law are shown in Figure S6. Notice that while the measured *C*_p_ is very close to the calculated limit for the Ag_2_Se-*x* wt % Bi_2_S_3_ composite and slightly below this limit for the pure Ag_2_Se sample, the limit is approximately within the error range of the measured values.^61,62^ The total thermal conductivity (*κ*_tot_) is determined by the equation
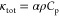
3where ρ is density. Figure 5a displays the obtained thermal conductivity of Ag_2_Se and Ag_2_Se-*x* wt % Bi_2_S_3_ samples over the whole temperature range. The pure Ag_2_Se pellet is characterized by a moderate κ_tot_ of 0.99 W m^–1^ K^–1^ at 300 K and 1.15 W m^–1^ K^–1^ at 390 K. These values are consistent with previous reports on Ag_2_Se.^25^ With the introduction of Bi_2_S_3_, κ_tot_ significantly decreases. The Ag_2_Se-1.5 wt % Bi_2_S_3_ sample displayed the lowest κ_tot_, 0.76 W m^–1^ K^–1^ at 300 K and 0.90 W m^–1^ K^–1^ at 390 K. Figure 5b displays the lattice thermal conductivity (κ_L_) obtained by subtracting the electronic contribution to the thermal conductivity calculated using a single parabolic band (SPB) model according to Wiedemann–Franz (κ_e_ = *L*σ*T*, where *L* is the Lorentz number) from the total thermal conductivity (κ_L_= κ_tot_ – κ_e_). The Lorenz number *L* is calculated by
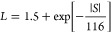
4The plot is displayed in Figure S7a. Figure S7b shows the temperature dependence of κ_e_. While similar κ_e_ values were obtained for the different materials, composites displayed lower κ_L_, down to 0.34–0.17 W m^–1^ K^–1^ for Ag_2_Se-1.5 wt % Bi_2_S_3_. The lower κ_tot_ measured for the composites is associated with a more effective scattering of phonons at point defects created by Bi^3+^ and extensive interphases between Ag_2_Se and Bi_2_S_3_ in the case of the highest doped samples. Numerous previous works have reported a decrease of thermal conductivity with a minor effect on electrical conductivity and have associated this phenomenon with different explanations, including a strong scattering on phonons created by precipitates without strongly affecting electrical conductivity,^63^ hierarchical architecture with multiscale defects differently affecting phonons and electrons,^60^ phonon scattering by introduced electrically dopant atoms,^64^ and preferential phonon scattering by introduced nanoparticles.^65^

**Figure 6:**
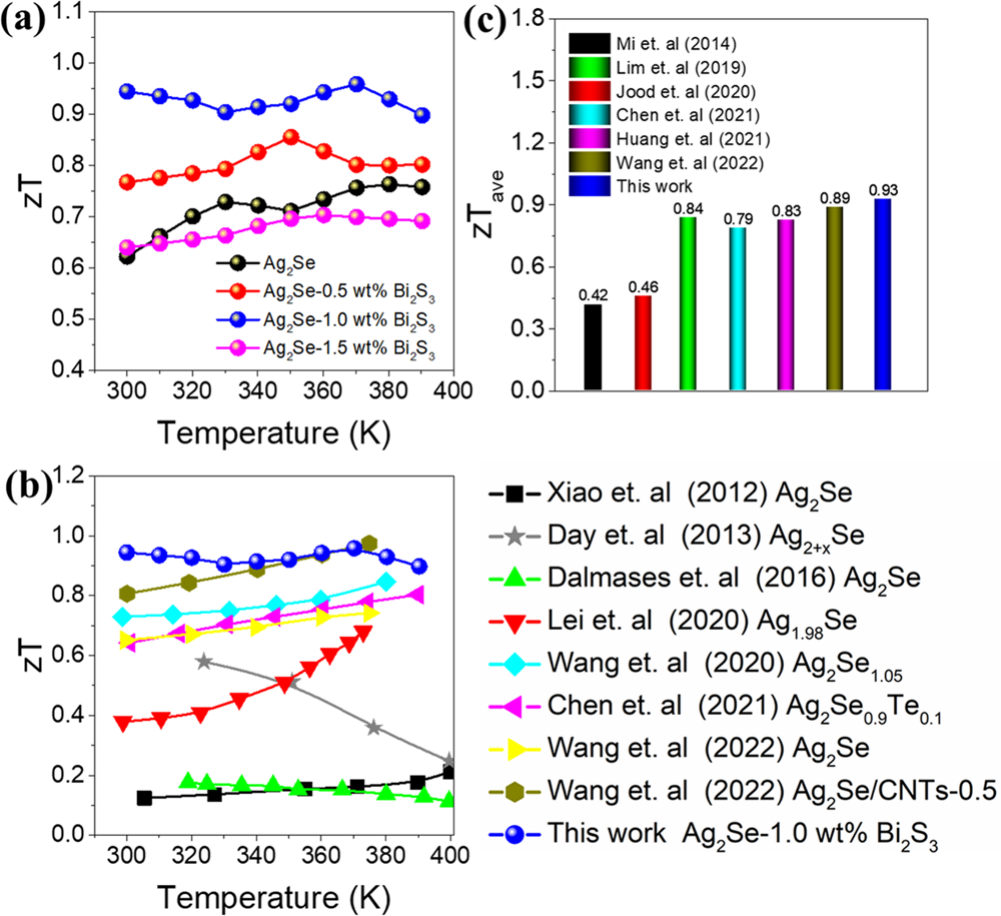
Temperature dependence of (a) *zT* values of Ag_2_Se-*x* wt % Bi_2_S_3_, (b) a comparison with reported silver selenide-based thermoelectric materials,^5,10,19,25,66,69,70^ and (c) a comparison of *zT*_ave_ with reported data of silver selenide-based thermoelectric materials.^5,11,13,15,18,68^

The temperature dependence of the TE figure or merit, *zT*, is displayed in Figure 6a. For the pristine Ag_2_Se pellet, the *zT* value increases from 0.62 at 300 K to 0.76 at 380 K. The *zT* values of the Ag_2_Se-*x* wt % Bi_2_S_3_ composites increase with the introduction of 0.5–1.0 wt % Bi_2_S_3_. A maximum *zT* value of 0.96 was obtained for the Ag_2_Se-1.0 wt % Bi_2_S_3_ sample at 370 K, which is ascribed to the highest PF value and slightly decreased κ_tot_. These *zT* values are above those previously reported n-type Ag_2_Se -based TE materials prepared by wet chemistry^18,19,22,23,25,27,66^ and other methods^15,32,67,68^ (Figure 5b and Table S5). We further determined the thermoelectric properties of a pure Ag_2_Se sample up to 480 K (Figure S8). We noticed that above 400 K, coinciding with the Ag_2_Se phase transition, a large decrease in the absolute value of the Seebeck coefficient and electrical conductivity was obtained, which resulted in an abrupt drop of *zT*. Thus, the material application is limited to a temperature range extending up to about 390 K.

*zT* values remain constant throughout the whole temperature range tested, providing a high average *zT* (*zT*_ave_) calculated as
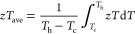
5where *T*_h_ is the hot-side temperature, *T*_c_ is the cold-side temperature, and *zT*_ave_ is thus the area under the *zT* curve divided by the value of *T*_h_ – *T*_c_. As shown in Figure 6c, a *zT*_ave_ = 0.93 is obtained in the temperature range of 300 to 390 K for the Ag_2_Se-1.0 wt % Bi_2_S_3_ sample, significantly above previously reported values.

## Conclusions

In conclusion, a facile, rapid, high-yield, and component-controllable room-temperature aqueous synthesis method was adopted to prepare a plethora of metal chalcogenide MX nanoparticles (M = Ag, Cu, Pb, Bi; X = S, Se). Using this procedure, a series of Ag_2_Se-*x* wt % Bi_2_S_3_ composites was obtained by blending the materials in solution and hot press sintering the obtained dried powder. A maximum *zT* value of 0.76 for pure Ag_2_Se was obtained at 380 K. Further investigation illustrates that moderate Bi_2_S_3_ doping can effectively increase the absolute S value and reduce κ_L_ without significant harm to σ, which contributes to a remarkable PF of 2.66 mW m^–1^ K^–2^ and a maximum *zT* of 0.96 at 370 K. Besides, a remarkable *zT*_ave_ of 0.93 was obtained for Ag_2_Se-1.0 wt % Bi_2_S_3_ nanocomposites, above the values obtained in most previous silver selenide-based thermoelectric materials fabricated via wet chemical approaches.

## Supporting Information Available

Experimental characterization details, additional SEM, XRD, EDX data, reproducibility results, heat capacities, calculation of the Lorenz number, and comparison with previous literature (PDF)
