# Supplementary material for: Engineering of Thermoelectric Composites Based on Silver Selenide in Aqueous Solution and Ambient Temperature
Source: ACS Appl Electron Mater. 2023 May 5;6(5):2807–15. doi: 10.1021/acsaelm.3c00055 (PMC11137807; doi:10.1021/acsaelm.3c00055)
Supplement: Supplementary file 1 — el3c00055_si_001.pdf [file el3c00055_si_001.pdf]

# Supporting Information

## Engineering of thermoelectric composites based on silver selenide in an aqueous solution and ambient temperature

Bingfei Nan [a, b], Mengyao Li [a, c], Yu Zhang [d] *, Ke Xiao [a, b], Khak Ho Lim [e, f], Cheng Chang [g, h], Xu Han [i], Yong Zuo [j], Junshan Li [k], Jordi Arbiol [i, l], Jordi Llorca [m], Maria Ibáñez [g], and Andreu Cabot [a, l] *

[a] Catalonia Institute for Energy Research - IREC, Sant Adrià del Besòs, Barcelona 08930, Spain

[b] Departament d'Enginyeria Electrònica i Biomèdica, Universitat de Barcelona, 08028 Barcelona, Catalonia, Spain

[c] School of Physics and Microelectronics, Zhengzhou University, Zhengzhou 450052, China

[d] Department of Materials Science and Engineering, Pennsylvania State University, Pennsylvania 16802, USA

[e] Institute of Zhejiang University- Quzhou, 99 Zheda Rd, Quzhou 324000, Zhejiang, P.R. China

[f] College of Chemical and Biological Engineering, Zhejiang University, 38 Zheda Rd, Hangzhou 310007, Zhejiang, P.R. China

[g] Institute of Science and Technology Austria, Am Campus 1, 3400 Klosterneuburg, Austria

[h] School of Materials Science and Engineering, Beihang University, Beijing 100191, China.

[i] Catalan Institute of Nanoscience and Nanotechnology (ICN2), Campus UAB, Bellaterra, 08193 Barcelona, Catalonia, Spain

[j] Istituto Italiano di Tecnologia, Via Morego 30, 16163 Genova, Italy

[k] Institute for Advanced Study, Chengdu University, 610106, Chengdu, China

[l] ICREA, Pg. Lluís Companys 23, 08010 Barcelona, Catalonia, Spain

[m] Institute of Energy Technologies, Department of Chemical Engineering and Barcelona Research Center in Multiscale Science and Engineering, Universitat Politècnica de Catalunya, EEBE, 08019 Barcelona, Catalonia, Spain

* E-mail addresses: yvz5897@psu.edu (Y. Zhang), acabot@irec.cat (A. Cabot).

## 1. Supporting experimental section

### 1.1. Structural and chemical characterizations

The NP morphology was characterized using a field emission scanning electron microscope (SEM, Zeiss Auriga) operated at 5.0 kV. The elemental composition ratios were analyzed via an energy dispersive X-ray spectrometer (EDX) inside the SEM at 20.0 kV. X-ray diffraction analyses (XRD) were carried out on a Bruker AXS D8 Advance X-ray diffractometer with Cu-Kα radiation ($\lambda$=1.5406 Å). Transmission electron microscopy (TEM), high resolution TEM (HRTEM), and scanning TEM (STEM) images were obtained on a FEI Tecnai F20 field emission gun microscope operating at 200 kV and equipped with high angle annular dark field (HAADF) and Gatan quantum electron energy loss spectroscopy (EELS) detectors.

### 1.2. Thermoelectric property measurements

The electrical resistivity and Seebeck coefficient of hot-pressed samples were measured automatically and simultaneously in a Linseis LSR-3 equipment under a helium atmosphere. All samples were measured at least three times under heating and cooling cycles processes. Considering the system-measurement accuracy, the error in the measurement of resistivity and Seebeck coefficient measurements was estimated to be ca. 5%. Thermal conductivity values of hot-pressed pellets were calculated according to $\kappa= \alpha\ C_p\ \rho$, where $\kappa$ is total thermal conductivity, $\alpha$ is thermal diffusivity ($mm^2\ s^{-1}$), $C_p$ is specific heat capacity ($J\ g^{-1}\ K^{-1}$), and $\rho$ is the density of pellets ($g\ cm^{-1}$). $\lambda$ was directly measured on a Linseis XFA 600 Xenon Flash apparatus with an estimated error of ca. 5%. $C_p$ values were calculated by Dulong–Petit approximation (3R law), and $C_p$ curves were also obtained in a Netzsch differential scanning calorimetry (DSC) at a heating rate of 5 °C/min in $N_2$ atmosphere. Relative $\rho$ values of the samples were estimated by the Archimedes' method. Hall carrier concentrations ($n_H$) and mobilities ($\mu_H$) at room temperature were measured using the Van der Pauw method and Hall Bar measurements (ezHEMS, NanoMagnetics) using a magnetic field of 1 T.

## 2. Supporting results

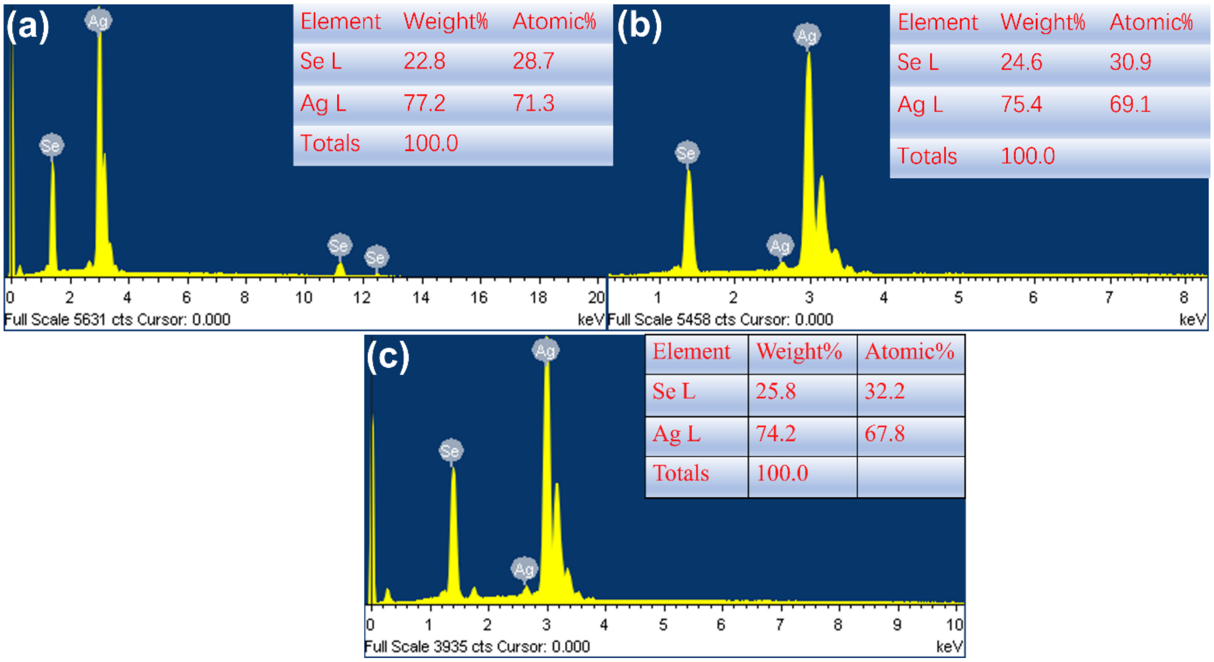


**Figure S1.** EDX spectra of the products obtained from the reaction between Ag and Se powders taken in the ratio (a) 2:1, (b) 1.9:1, (c) 1.8:1.

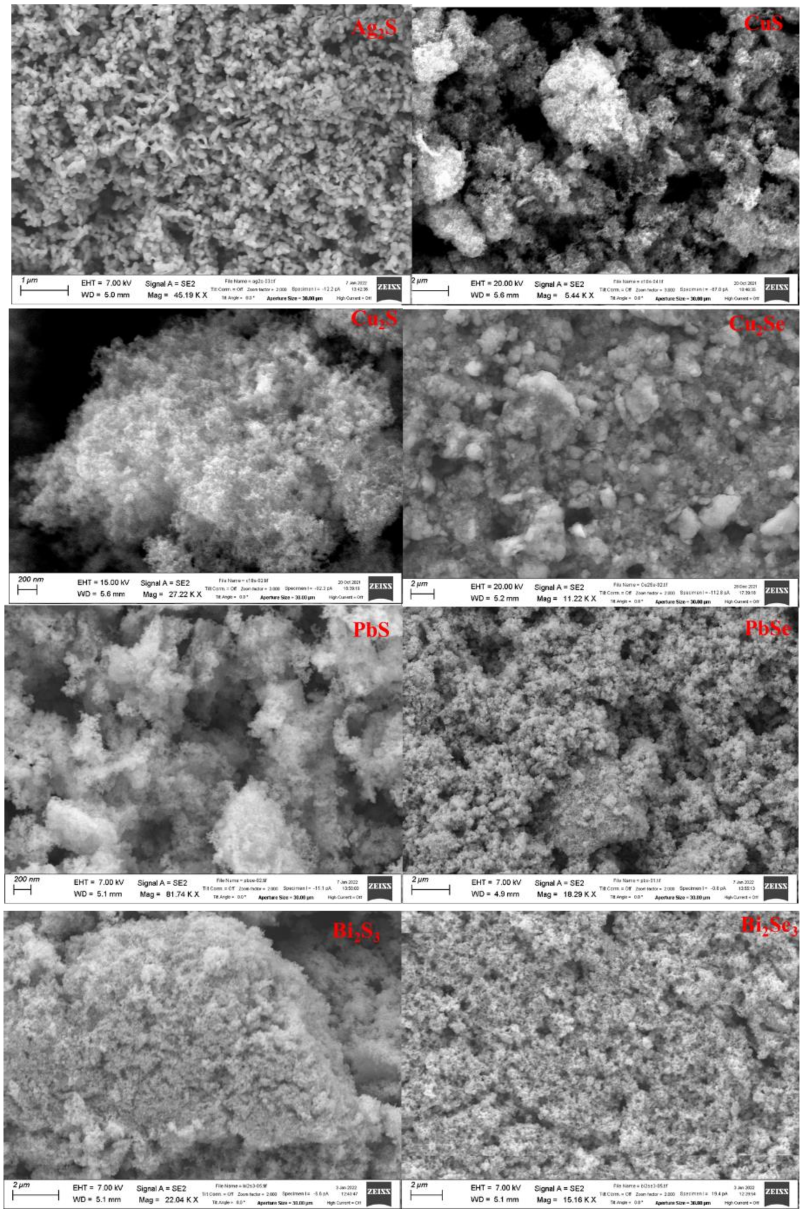


**Figure S2**. SEM images of MX NPs (M=Ag, Cu, Pb and Bi, X=S and Se).

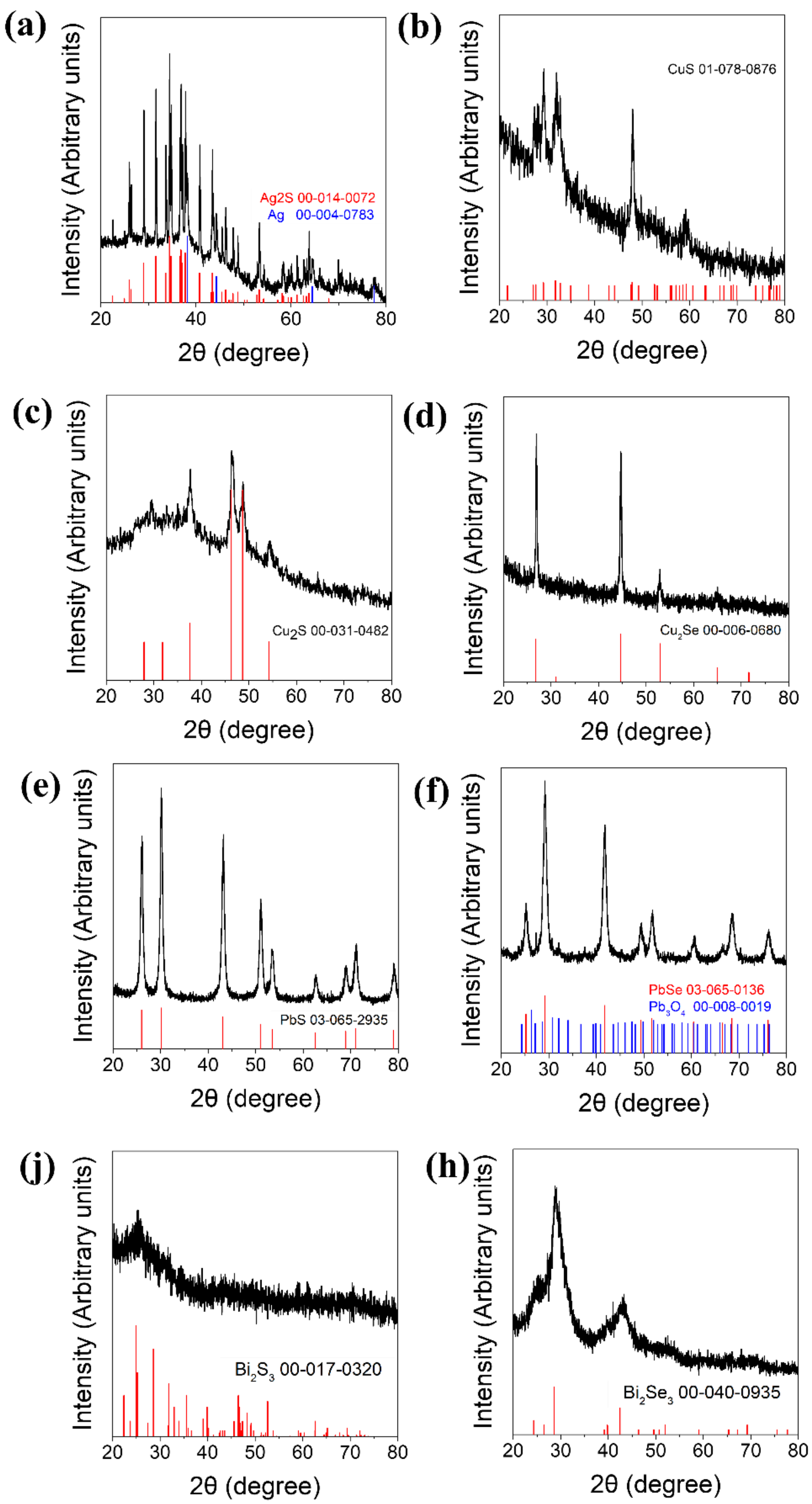


**Figure S3**. XRD patterns of MX NPs (M=Ag, Cu, Pb and Bi, X=S and Se): (a) $Ag_2S$, (b) CuS, (c) $Cu_2S$, (d) $Cu_2Se$, (e) PbS, (f) PbSe, (j) $Bi_2S_3$ and (h) $Bi_2Se_3$.

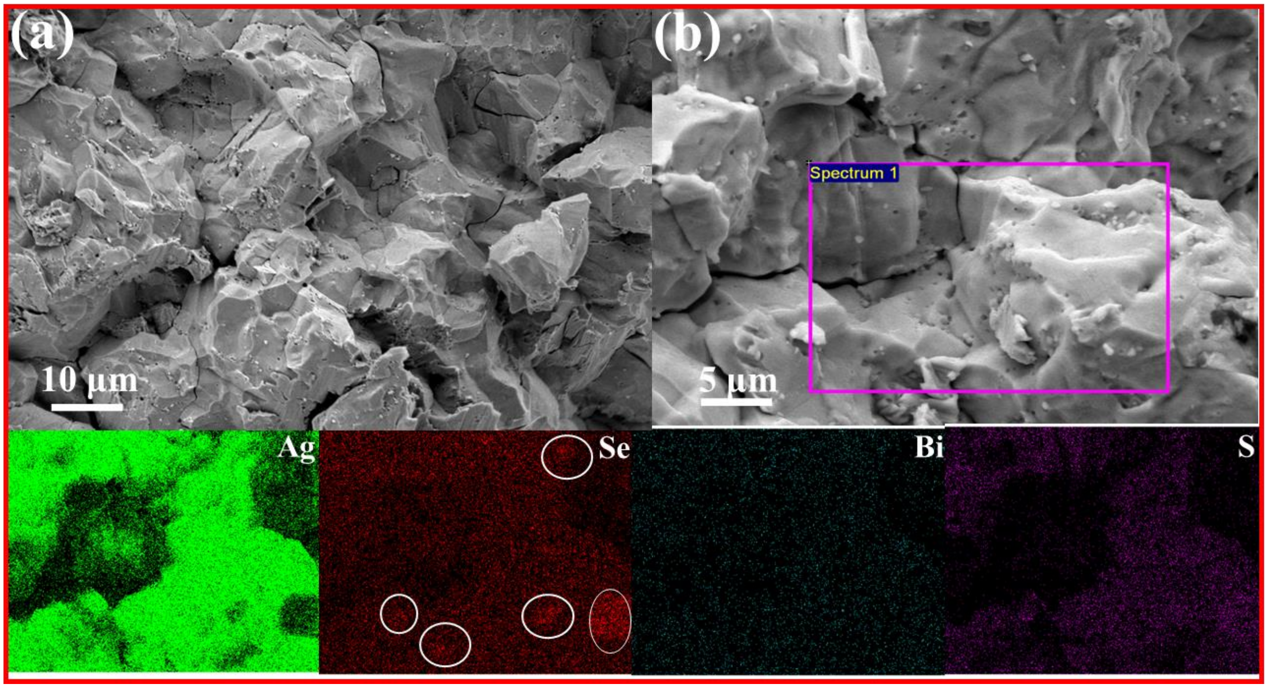


**Figure S4.** (a-b) Cross-section SEM micrographs of $Ag_2Se$-1.0 wt% $Bi_2S_3$ pellet and corresponding compositional maps of Ag, Se, Bi and S from the spectrum 1 in the Figure b (Se-rich regions marked with white circles).

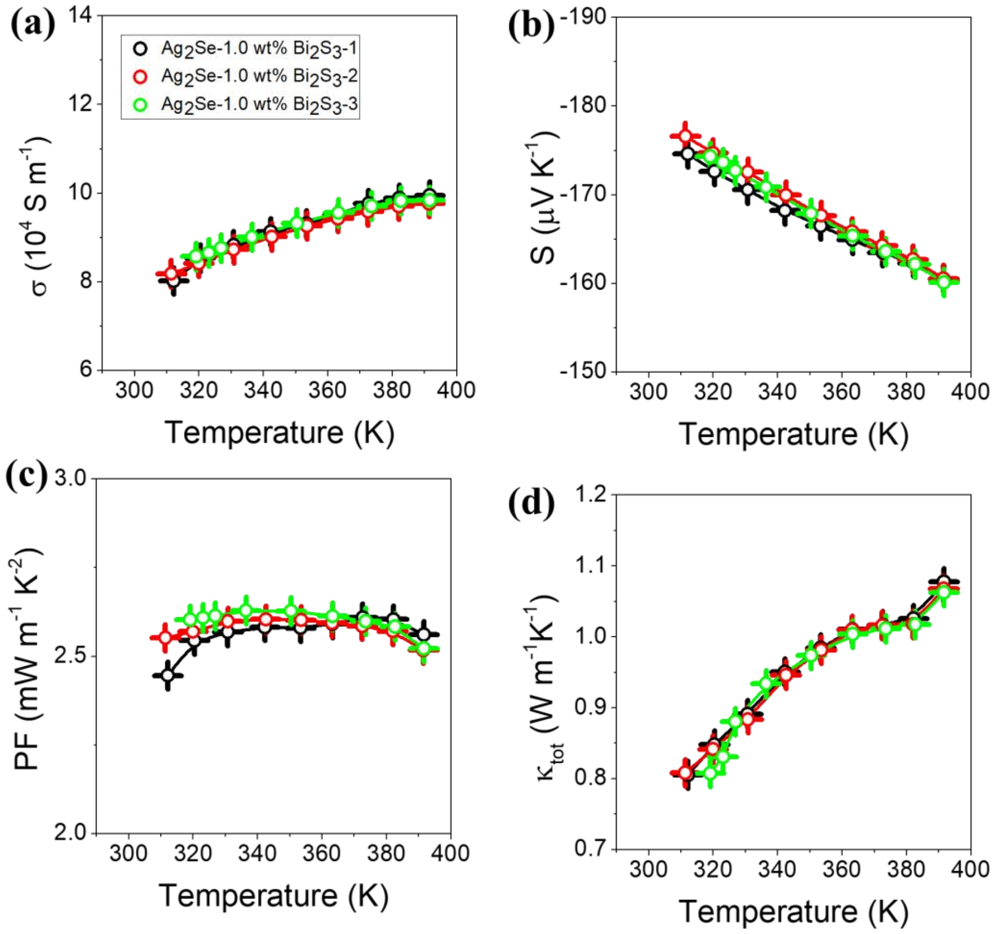


**Figure S5**. **Reproducibility**: temperature dependence of (a) electrical conductivity, σ; (b) Seebeck coefficient, S; (c) power factor, PF; (d) total thermal conductivity, $\kappa_{tot}$ of $Ag_2Se$ -1.0 wt% $Bi_2S_3$ pellets measured three consecutive up-down cycles during the heating up from room temperature to 390 K.

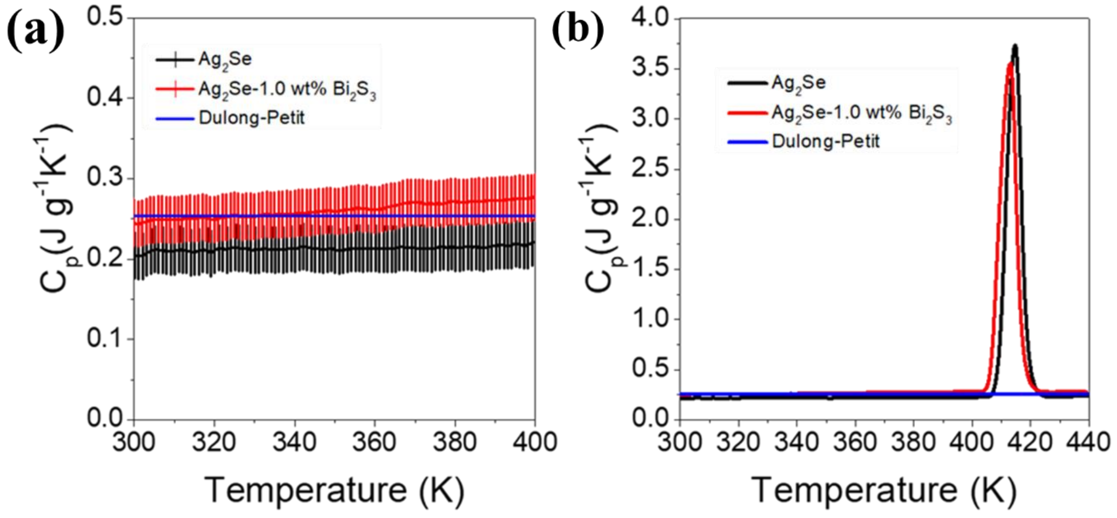


**Figure S6**. (a) Dulong-Petit specific heat capacity ($C_p$) of $Ag_2Se$ pellet and experimental temperature dependence of $C_p$ of $Ag_2Se$ and $Ag_2Se$ -1.0 wt% $Bi_2S_3$ pellets. (b) The calculated $C_p$ from the Dulong-Petit approximation (blue line) is 0.254 J $g^{-1}$K.

Round-robin studies indicate that the error in the measurement of Cp can be as high as between 5 and 10%. Thus we included 10% error bars in our experimental data in Figure S6. Besides, notice that Dulong-Petit's law states that the molar-specific heat capacity of a solid element or compound at constant volume is approximately equal to 3R, where R is the molar gas constant. This law is valid for elements and compounds at high temperatures, where the vibrational degrees of freedom of the atoms are excited. Dulong-Petit's may fail at low temperatures due to higher-energy vibrational modes not being populated. [1, 2] In the case of $Ag_2Se$-1.0 wt% $Bi_2S_3$, the measured specific heat becomes slightly higher than Dulong-Petit's limit at temperatures higher than 330 K. This can be attributed to the presence of defects and impurities, particularly $Bi^{3+}$ ions, in the $Ag_2Se$ crystal structure that can act as additional vibrational modes and contribute to the specific heat. Furthermore, the lattice vibrations of the crystal may be affected by the presence of defects, resulting in higher specific heat. Notice that several other works have also shown Cp similar to and even below Dulong Petit's limit.[3-5] Notice in addition, that the phase transition temperature decreases slightly after the introduction of 1.0 wt% $Bi_2S_3$, which is consistent with the phenomenon observed in $Cu_2Se$ alloyed with $Ag_2Se$. [6]

### Calculation of Lorenz number

Here, the Lorenz number L is calculated based on the measured Seebeck coefficient. So we used the equation [7]:

$$L = 1.5 + exp\left[-\frac{|S|}{116}\right] \qquad \text{(S1)}$$

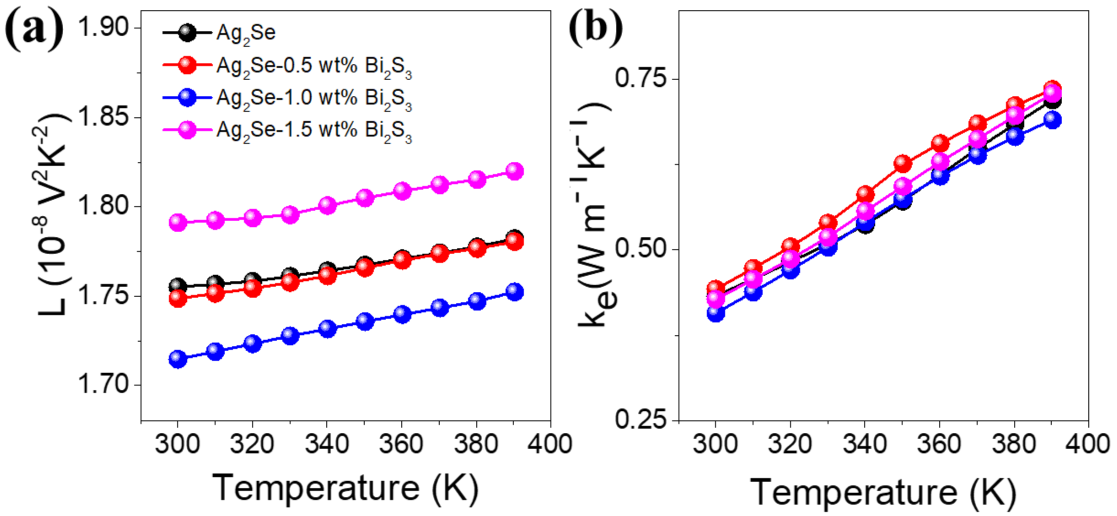


**Figure S7**. Temperature dependence of (a) Lorentz number (L) and (b) $\kappa_e$ of $Ag_2Se$ -x wt% $Bi_2S_3$ samples (x=0, 0.5, 1.0 and 1.5)

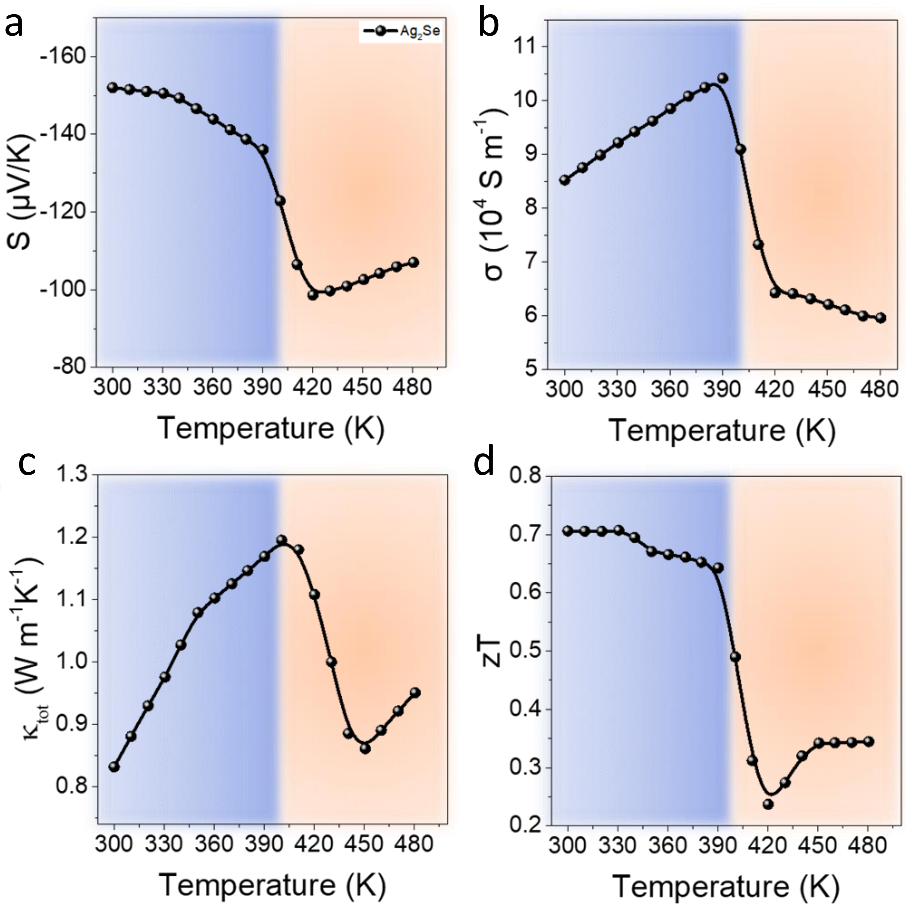


**Figure S8.** Thermoelectric properties of the $Ag_2Se$ pellet across phase transition. (a) Seebeck coefficient, S; (b) electrical conductivity, σ; (c) total thermal conductivity, $\kappa_{tot}$ and (d) figure of merit, zT.

As shown in Figure S8, above 400 K, when the phase transition of $Ag_2Se$ takes place, the Seebeck coefficient (S) of the pure $Ag_2Se$ significantly decreases and then slightly rises. Above 400 K, the electrical conductivity (σ) values continue to decrease to $5.96\times10^4$ S $m^{-1}$. Therefore, the zT curve shows a sharp decrease at the phase transition temperature, consistent with previous reports.[8, 9] This result highlights the significant deterioration of the TE performance of $Ag_2Se$ at higher temperatures after the phase transition. Therefore, careful selection of the operating temperature range is crucial when using $Ag_2Se$ for thermoelectric applications.

**Table S1.** Synthesis of binary metal chalcogenides MX nanoparticles (M=Ag, Cu, Pb and Bi, Sn, X=S and Se).

| Nanomaterial | M (solvent: $H_2O$) | X (dissolved into 5 mL) $N_2H_4$ $H_2O$) | Time |
|---|---|---|---|
| $Ag_2S$ | 10 mL 0.5 M $AgNO_3$ | 2.5 mmol S | Instant |
| CuS | 10 mL 0.25 M $Cu(NO_3)_2 \cdot 3H_2O$ | 2.5 mmol S | Instant |
| $Cu_2S$ | 10 mL 0.5 M $Cu(NO_3)_2 \cdot 3H_2O$ | 2.5 mmol S | Instant |
| $Cu_2Se$ | 10 mL 0.5 M $Cu(NO_3)_2 \cdot 3H_2O$ | 2.5 mmol Se | Instant |
| PbS | 10 mL 0.25 M $Pb(NO_3)_2$ | 2.5 mmol S | Instant |
| PbSe | 10 mL 0.25 M $Pb(NO_3)_2$ | 2.5 mmol Se | Instant |
| $Bi_2S_3$ | 10 mL 0.167 M $Bi(NO_3)_3 \cdot 5H_2O$ + 2 mL concentrated $HNO_3$ | 2.5 mmol S | Instant |
| $Bi_2Se_3$ | 10 mL 0.167 M $Bi(NO_3)_3 \cdot 5H_2O$ + 2 mL concentrated $HNO_3$ | 2.5 mmol Se | Instant |
| SnS | 2.5 mmol $SnCl_2$ + 4 M 20 mL NaOH | 2.5 mmol S | ca. 1 h |
| SnSe | 2.5 mmol $SnCl_2$ + 4 M 20 mL NaOH | 2.5 mmol Se | ca. 1 h |

Note: based on the electrochemical theory, $Te^0$ element cannot be efficiently reduced to $Te^{2-}$ ions by $N_2H_4$ $H_2O$ due to the proximity of redox potential ($E^0_{\mathrm{Te/Te^{2-}}}$= -1.143 V and $E^0_{N_2/N_2H_4}$= -1.143V).[10]

**Table S2**. EDS test of MX NPs (M=Ag, Cu, Pb and Bi, X=S and Se) (Atomic%).

| Sample | $M^{n+}$ | S/Se |
|---|---|---|
| $Ag_2S$ | 33.7 | 66.3 |
| CuS | 50.5 | 49.5 |
| $Cu_2Se$ | 63.2 | 36.8 |
| PbS | 47.1 | 52.9 |
| PbSe | 49.0 | 51.0 |
| $Bi_2S_3$ | 39.5 | 60.5 |
| $Bi_2Se_3$ | 40.3 | 59.7 |
| SnS | 51.7 | 48.3 |
| SnSe | 53.9 | 46.1 |

**Table S3.** EDS compositions from the cross-section SEM of $Ag_2Se$ -x wt% $Bi_2S_3$ pellets (atomic%).

| Samples | Ag | Se | Bi | S |
|---|---|---|---|---|
| $Ag_2Se$ | 69 | 31 | 0 | 0 |
| $Ag_2Se$ -0.5 wt% $Bi_2S_3$ | 67 | 32 | 0.2 | 0.3 |
| $Ag_2Se$ -1.0 wt% $Bi_2S_3$ | 63 | 36 | 0.4 | 0.6 |
| $Ag_2Se$ -1.5 wt%B $Bi_2S_3$ | 66 | 33 | 0.6 | 0.9 |

**Table S4**. Experimental density (ρ), relative density($\rho_r$), and thermal diffusivity（α) of hot-pressed $Ag_2Se$-x wt% $Bi_2S_3$ pellets (room temperature).

| Samples | ρ ($g/cm^3$) | $\rho_r$(%) | α ($mm^2/s$) |
|---|---|---|---|
| $Ag_2Se$ | 7.71 | 93.8% | 0.51 |
| $Ag_2Se$ -0.5 wt% $Bi_2S_3$ | 7.68 | 93.5% | 0.44 |
| $Ag_2Se$ -1.0 wt% $Bi_2S_3$ | 7.57 | 92.1% | 0.42 |
| $Ag_2Se$ -1.5 wt% $Bi_2S_3$ | 7.55 | 91.9% | 0.40 |

**Table S5**. Comparison of TE properties of $Ag_2Se$-based materials.

| Samples | Preparation method | zT | Temperature (K) | Year [Refs.] |
|---|---|---|---|---|
| $Ag_2Se$<br>$Ag_4SeS$ | colloidal synthesis | 0.23<br>0.33 | 408<br>355 | 2012 [11] |
| $Ag_2Se_{1.08}$ | solid phase | 0.96 | 401 | 2014[8] |
| $Ag_2Se$ | manual mixing | 0.8 | 390 | 2016 [12] |
| $Ag_2Se$ | hand-mixing and grinding | 1.2 | 390 | 2017 [13] |
| $Ag_2Se$+5 mol% of Te | colloidal synthesis | 0.89 | 343 | 2019 [14] |
| $(Ag_2Se)_{1-x}$(nano-$Cu_2Se)_x$ | Melting | 0.45 | 875 | 2019 [6] |
| $Ag_2Se$ | aqueous synthesis | 0.84 | 380 | 2020[9] |
| $Ag_2Se$<br>$Ag_{1.9}Sn_{0.1}Se$ | wet chemical method | 0.7<br>0.9 | 317<br>300 | 2020[15] |
| β-$Ag_2Se$ | ball milling | ~0.7<br>~0.9 | 300<br>390 | 2020 [16] |
| $Ag_{1.98}Se$ | solid phase | 0.68 | 373 | 2020 [17] |
| $Ag_2Se$ | zone-melting | 0.75 | 300 | 2021 [18] |
| $Ag_2Se$ | liquid-state manipulation | 1.21 | 389 | 2021 [19] |
| $(Ag_{1-x}Cu_x)_2Se$ | mechanical alloying | 0.79 | 300–380 | 2021[20] |
| $Ag_2Se$/CNTs-x | in situ solution synthesis | 0.97 | 375 | 2021 [21] |
| $Ag_2Se$/MWCNTs | hydrothermal method | 0.07 | 400 | 2022 [22] |
| $Ag_2Se/AgSbSe_2$ | solution-synthesized mixture of $Ag_2Se$ and $Sb_2Se_3$ | 0.93 | 377 | 2023 [23] |
| $Ag_2Se$/ZnSe | ball milling | ~0.7 | 383 | 2023 [24] |
| **$Ag_2Se$-1.0 wt%$Bi_2S_3$** | **aqueous synthesis** | **0.96** | **370** | **This work** |

## REFERENCES


(1) Simon, Steven H. The Oxford Solid State Basics. 2013, OUP Oxford. ISBN 978-0-19-968076-4

(2) Landau, L. D.; Lifshitz, E. M. Statistical Physics Pt. 1. Course in Theoretical Physics. 1980, Vol. 5 (3rd ed.). Oxford: Pergamon Press. p. 193,196

(3) Zhang, Y.; Liu, Y.; Lim, K. H.; Xing, C.; Li, M.; Zhang, T.; Tang, P.; Arbiol, J.; Llorca, J.; Ng, K. M.; Ibáñez, M.; Guardia, P.; Prato, M.; Cadavid, D.; Cabot, A. Tin Diselenide Molecular Precursor for Solution-Processable Thermoelectric Materials. *Angew.Chem. Int. Ed.* **2018**, *130*, 17309-17314.

(4) Saha, B.; Acharya, J.; Sands, T. D.; Waghmare, U. V. Electronic structure, phonons, and thermal properties of ScN, ZrN, and HfN: A first-principles study. *J. Appl. Phys.* **2010**, *107*, 033715.

(5) Li, M. Y.; Liu, Y.; Zhang, Y.; Han, X.; Zhang, T.; Zuo, Y.; Xie, C. Y.; Xiao, K.; Arbiol, J.; Llorca, J.; Ibanez, M.; Liu, J. F.; Cabot, A. Effect of the Annealing Atmosphere on Crystal Phase and Thermoelectric Properties of Copper Sulfide. *ACS Nano* **2021**, *15*, 4967-4978.

(6) Ballikaya, S.; Oner, Y.; Temel, T.; Ozkal, B.; Bailey, T. P.; Toprak, M. S.; Uher, C. Thermoelectric and thermal stability improvements in Nano-$Cu_2Se$ included $Ag_2Se$. *J. Solid State Chem.* **2019**, *273*, 122-127.

(7) Mallick, M. M.; Rösch, A. G.; Franke, L.; Gall, A.; Ahmad, S.; Geßwein, H.; Mazilkin, A.; Kübel, C.; Lemmer, U. New frontier in printed thermoelectrics: formation of β-$Ag_2Se$ through thermally stimulated dissociative adsorption leads to high ZT. *J. Mater. Chem. A* **2020**, *8*, 16366-16375.

(8) Mi, W.; Qiu, P.; Zhang, T.; Lv, Y.; Shi, X.; Chen, L. Thermoelectric transport of Se-rich $Ag_2Se$ in normal phases and phase transitions. *Appl. Phys. Lett.* **2014**, *104*, 133903.

(9) Wang, H.; Liu, X.; Zhang, B.; Huang, L.; Yang, M.; Zhang, X.; Zhang, H.; Wang, G.; Zhou, X.; Han, G. General surfactant-free synthesis of binary silver chalcogenides with tuneable thermoelectric properties. *Chem. Eng. J.* **2020**, *393*, 124763.

(10) Wan, B.; Hu, C.; Feng, B.; Xi, Y.; He, X. Synthesis and thermoelectric properties of PbTe nanorods and microcubes. *Mater. Sci. Eng. B* **2009**, *163*, 57-61.

(11) Xiao, C.; Xu, J.; Li, K.; Feng, J.; Yang, J.; Xie, Y. Superionic Phase Transition in Silver Chalcogenide Nanocrystals Realizing Optimized Thermoelectric Performance. *J. Am. Chem. Soc.* **2012**, *134*, 4287-4293.

(12) Duan, H. Z.; Li, Y. L.; Zhao, K. P.; Qiu, P. F.; Shi, X.; Chen, L. D. Ultra-Fast Synthesis for $Ag_2Se$ and CuAgSe Thermoelectric Materials. *JOM* **2016**, *68*, 2659-2665.

(13) Yang, D.; Su, X.; Meng, F.; Wang, S.; Yan, Y.; Yang, J.; He, J.; Zhang, Q.; Uher, C.; Kanatzidis, M. G.; Tang, X. Facile room temperature solventless synthesis of high thermoelectric performance $Ag_2Se$ via a dissociative adsorption reaction. *J. Mater. Chem. A* **2017**, *5*, 23243-23251.

(14) Lim, K. H.; Wong, K. W.; Liu, Y.; Zhang, Y.; Cadavid, D.; Cabot, A.; Ng, K. M. Critical role of nanoinclusions in silver selenide nanocomposites as a promising room temperature thermoelectric material. *J. Mater. Chem. C* **2019**, *7*, 2646-2652.

(15) Li, D.; Zhang, J. H.; Li, J. M.; Zhang, J.; Qin, X. Y. High thermoelectric performance for an $Ag_2Se$-based material prepared by a wet chemical method. *Mater. Chem. Front.* **2020**, *4*, 875-880.

(16) Chen, J.; Sun, Q.; Bao, D.; Liu, T.; Liu, W.-D.; Liu, C.; Tang, J.; Zhou, D.; Yang, L.; Chen, Z.-G. Hierarchical Structures Advance Thermoelectric Properties of Porous n-type β-$Ag_2Se$. *ACS Appl. Mater. Interfaces* **2020**, *12*, 51523-51529.

(17) Lei, Y.; Liu, W.; Zhou, X.; Luo, J.; Zhang, C.; Su, X.; Tan, G.; Yan, Y.; Tang, X. The electronic-thermal transport properties and the exploration of magneto-thermoelectric properties and the Nernst thermopower of $Ag_{2(1+x)}Se$. *J. Solid State Chem.* **2020**, *288*, 121453.

(18) Jin, M.; Liang, J.; Qiu, P.; Huang, H.; Yue, Z.; Zhou, L.; Li, R.; Chen, L.; Shi, X. Investigation on Low-Temperature Thermoelectric Properties of $Ag_2Se$ Polycrystal Fabricated by Using Zone-Melting Method. *J. Phys. Chem. Lett.* **2021**, *12*, 8246-8255.

(19) Li, D.; Zhang, B. L.; Ming, H. W.; Wang, L.; Zu, Y.; Qin, X. Y. Liquid-Phase Manipulation Securing Enhanced Thermoelectric Performance of $Ag_2Se$. *ACS Appl. Mater. Interfaces* **2021**, *13*, 34543−34549.

(20) Chen, J.; Sun, Q.; Bao, D.; Tian, B.-Z.; Wang, Z.; Tang, J.; Zhou, D.; Yang, L.; Chen, Z.-G. Simultaneously enhanced strength and plasticity of $Ag_2Se$-based thermoelectric materials endowed by nano-twinned CuAgSe secondary phase. *Acta Mater.* **2021**, 117335.

(21) Wang, H. T.; Ma, H. Q.; Duan, B.; Geng, H. Y.; Zhou, L.; Li, J. L.; Zhang, X. L.; Yang, H. J.; Li, G. D.; Zhai, P. C. High-Pressure Rapid Preparation of High-Performance Binary Silver Sulfide Thermoelectric Materials. *ACS Appl. Energy Mater.* **2021**, *4*, 1610-1618.

(22) Chen, N.; Ren, C.; Sun, L.; Xue, H.; Yang, H.; An, X.; Yang, X.; Zhang, J.; Che, P. Improved thermoelectric properties of multi-walled carbon nanotubes/$Ag_2Se$ via controlling the composite ratio. *CrystEngComm* **2022**, *24*, 260-

268.
(23) Wang, H.; Han, G.; Zhang, B.; Chen, Y.; Liu, X.; Zhang, K.; Lu, X.; Wang, G.; Zhou, X. $AgSbSe_2$ Inclusions Enabling High Thermoelectric and Mechanical Performance in n-Type $Ag_2Se$-based Composites. *Acta Materialia* **2023**, 118753.
(24) Feng, B.; Cheng, Y.; Liu, C.; Gao, J.; Wu, G.; Bai, X.; Si, R.; Li, W.; Guo, Y.; Miao, L. Ag Interstitial Inhibition and Phonon Scattering at the ZnSe Nano-Precipitates to Enhance the Thermoelectric Performance of $Ag_2Se$. *ACS Appl. Energy Mater.* **2023**, *6*, 2804−2811.
